# A synbio approach for selection of highly expressed gene variants in Gram-positive bacteria

**DOI:** 10.1186/s12934-018-0886-y

**Published:** 2018-03-08

**Authors:** Roberto Ferro, Maja Rennig, Cristina Hernández-Rollán, Daniel O. Daley, Morten H. H. Nørholm

**Affiliations:** 1 Novo Nordisk Foundation Center for Biosustainability, Technical University of Denmark, 2800 Kgs. Lyngby, Denmark; 2 Department of Plant and Environmental Science, University of Copenhagen, 1871 Frederiksberg, Denmark; 3 Center for Biomembrane Research, Department of Biochemistry and Biophysics, Stockholm University, Stockholm, Sweden; 4 CloneOpt AB, Upplands Väsby, Sweden

## Abstract

**Background:**

The market for recombinant proteins is on the rise, and Gram-positive strains are widely exploited for this purpose. *Bacillus subtilis* is a profitable host for protein production thanks to its ability to secrete large amounts of proteins, and *Lactococcus lactis* is an attractive production organism with a long history in food fermentation.

**Results:**

We have developed a synbio approach for increasing gene expression in two Gram-positive bacteria. First of all, the gene of interest was coupled to an antibiotic resistance gene to create a growth-based selection system. We then randomised the translation initiation region (TIR) preceding the gene of interest and selected clones that produced high protein titres, as judged by their ability to survive on high concentrations of antibiotic. Using this approach, we were able to significantly increase production of two industrially relevant proteins; sialidase in *B. subtilis* and tyrosine ammonia lyase in *L. lactis.*

**Conclusion:**

Gram-positive bacteria are widely used to produce industrial enzymes. High titres are necessary to make the production economically feasible. The synbio approach presented here is a simple and inexpensive way to increase protein titres, which can be carried out in any laboratory within a few days. It could also be implemented as a tool for applications beyond TIR libraries, such as screening of synthetic, homologous or domain-shuffled genes.

**Electronic supplementary material:**

The online version of this article (10.1186/s12934-018-0886-y) contains supplementary material, which is available to authorized users.

## Background

The advent of recombinant protein technology has enabled commercial applications for biopharmaceutical proteins and industrial biocatalysts not possible when the only option was to extract proteins from their original hosts. The enzyme market in particular has bloomed as a result of the production costs approaching those of the chemical industry [1]. To achieve low costs, enzymes are produced in large quantities by exploiting cell factories and large-scale bioreactors, but continuous improvements in the design of cell factories are needed to keep the production competitive and open markets for new products [2].

Various rational engineering approaches for cell factories are routinely employed to improve production. In the initial process a suitable expression host needs to be selected. Despite the documented role of *Escherichia coli* in molecular biology, other bacterial expression systems have been explored in biotechnology, taking advantage of divergent metabolism, secretion capability and biosafety of their protein-based products [3, 4]. Amongst them, various Gram-positive bacteria are of great interest due to e.g. their highly efficient protein secretion and GRAS status. *Bacillus subtilis* is routinely used industrially for its ability to secrete large amount of enzymes, which simplifies protein recovery and purification, leading to yields up to 20–25 g/L [5]. *Lactococcus lactis* has a long history of use in food microbiology and in the dairy industry [6], and it has lately risen as an alternative for production of membrane proteins [7, 8], secreted proteins [9, 10] and plant-based proteins and secondary metabolites [11–13].

Previously we have shown that protein production in a Gram-negative bacterium can be significantly increased by synthetically evolving a part of the translation initiation region (TIR) [14]. In expression clones the TIR extends from the region upstream of the Shine-Dalgarno (SD) sequence to the 5th or 6th codon of the gene of interest [15]. Whilst most TIRs function, they can support higher production titres if they are evolved with an appropriate selection pressure—for example by creating large libraries of randomised TIRs and selecting one that produces the most protein [16, 17].

Yet screening approaches for those libraries are limited. High throughput screening methods like fluorescence activated cell sorting (FACS) or droplet microfluidics enable the assessment of large libraries [18–20] but their use is restricted to phenotypes associated with a fluorophore to effectively screen for e.g. increased protein production [21]. Fusions with reporter proteins, such as fluorescent proteins, can also compromise the expression, solubility and bioactivity of a protein and are not suitable in industrial set-ups [22, 23]. The availability of other types of biosensors is limited and developing one for a new target is a laborious and time consuming process [24].

**Fig. 1:**
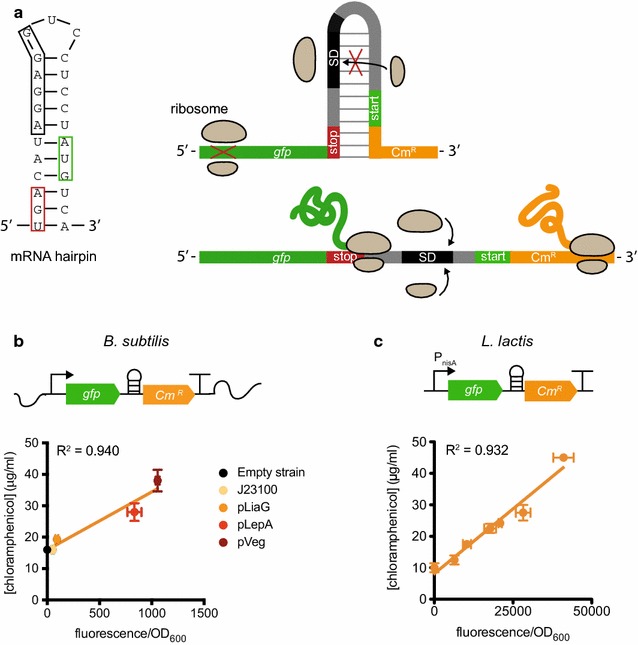
Testing of a translational coupling device in *Bacillus subtilis* and *Lactococcus lactis*. **a** To asses the efficacy of a specific translational coupling device in *B. subtilis* and *L. lactis* an mRNA hairpin structure was sandwiched between the gene encoding green fluorescent protein (*gfp*, green) and the chloramphenicol resistance gene (Cm^R^, orange). Left side: the predicted structure of the translational coupling device. Presumably, the stem of the mRNA hairpin structure consists of 11 nucleotide pairs comprising the stop codon of the upstream gene (red box) and the start codon of the downstream gene (green box). The ribosome binding site (black box) of the downstream gene is designed to be masked by the secondary mRNA structure. Upper right side: when the upstream gene (green, *gfp*) is not translated, the mRNA hairpin structure will not be resolved and the ribosome binding site of the downstream gene (orange, Cm^R^) remains inaccessible for the ribosome. Therefore, there is no translation of the downstream gene. Lower right side: when a ribosome translates *gfp*, the ribosome’s helicase activity will melt the secondary mRNA structure which makes the ribosome binding site accessible and the chloramphenicol resistance gene can be translated. The correlation between protein production, determined as fluorescence normalized for cell density, and chloramphenicol resistance, determined as minimal inhibitory concentration (MIC), was determined for genome-based expression in *B. subtilis* (**b**) and for plasmid-based expression in *L. lactis* (**c**)

For this purpose, we have established a phenotypic screening approach for TIR libraries in *E. coli* [25]. The approach utilises an antibiotic resistance gene that is translationally coupled to the gene of interest. In the bicistronic mRNA design, a hairpin-like structure separates an upstream gene of interest from a downstream antibiotic selection marker, thereby sequestering the SD site of the latter. Only upon efficient translation of the upstream gene, antibiotic resistance is obtained because the helicase activity of the translating ribosome allows expression of the downstream resistance gene (Fig. 1a). In this study we set out to determine if a similar synbio approach for optimising and selecting TIRs, would lead to increased production levels in industrially relevant Gram-positive bacteria.

## Results

### Characterization of a translational coupling device in Gram-positive hosts

We first set out to explore the applicability of a translational coupling design that was previously developed in *E. coli* [25]. To this end we constructed a plasmid for *L. lactis* and *B. subtilis* that contained the gene encoding green fluorescent protein (*gfp*), coupled by a sequence with hairpin-forming (hp) propensity to a chloramphenicol resistance gene (*gfp*-*hp*-*Cm*^*R*^) (Fig. 1a).

The *gfp*-*hp*-*Cm*^*R*^ construct was expressed from the nisin-inducible promoter P_nisA_ in a pNZ8048 vector in *L. lactis* [26]. In *B. subtilis* a set of four different constitutive promoters of increasing strength (P_J23101_, P_liaG_, P_lepA_ and P_veg_ [27]) were used and the constructs were integrated into the *amyE* locus on the chromosome [28]*. L. lactis* cultures were grown overnight, diluted in the morning and induced with variable concentrations of nisin (0.25–10 ng/mL); *B. subtilis* cultures were treated the same way, but did not require induction. Fluorescence was measured when the cultures reached late exponential phase and was normalized by cell density. At the same time resistance was assessed by plating on different concentrations of chloramphenicol. In both *L. lactis* and *B. subtilis* fluorescence levels showed a linear correlation (R^2^ > 0.9) with resistance to chloramphenicol, the latter measured as the minimum inhibitory concentration (MIC) (Fig. 1b, c). This demonstrates that the translational coupling device in combination with a chloramphenicol resistance gene can work over a broad range as a reporter of gene expression in these two Gram-positive model bacteria.

### Characterization of translation initiation region libraries in *L. lactis* and *B. subtilis*

**Fig. 2:**
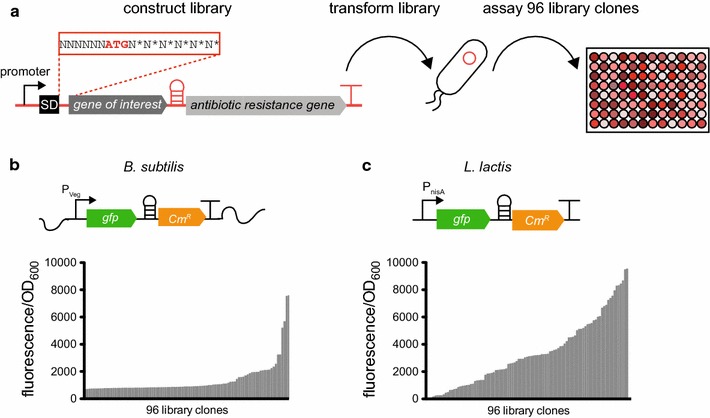
A part of the translation initiation region (TIR) affects expression in *Bacillus subtilis* and *Lactococcus lactis*. **a** TIR libraries (NNNNNNATGN*N*N*N*N*N*, see main text for further details) were constructed by PCR, transformed into the host strains and individual library clones were grown to asses the expression level ranges in *B. subtilis* and *L. lactis*. Fluorescence normalized by cell density was determined for 96 library clones for *B. subtilis* (**b**) and *L. lactis* (**c**). The *B. subtilis* library was expressed from the genome whereas the *L. lactis* library was expressed from a plasmid

Next we introduced sequence diversity in a specific part of the translation initiation region (TIR)—an approach that has proven successful for expression optimization in *E. coli* [14]. In this experiment the six nucleotides upstream of the start codon are randomized and the second and third codon of the open reading frame are concurrently substituted with synonymous codons (Fig. 2a). Depending on the nature of the 2nd and 3rd amino acid in the protein sequence, this system generates a maximum library size of about 150,000 variants and up to 1000-fold variation in gene expression levels in *E. coli* [14].

In the case of *gfp*, a library with about 50,000 variants was constructed in both hosts. In *B. subtilis* we chose a strong constitutive promoter (P_veg_) to control expression of *gfp*. The library was integrated into the *amyE* locus on the chromosome using an integrative vector propagated in *E. coli*. In *L. lactis* we used the pNZ8048 plasmid with the inducible nisin promoter controlling the expression of *gfp*. Both libraries were based on the constructs used for the initial characterization of the translational coupling device *gfp*-*hp*-*Cm*^*R*^. Sequence variation was introduced by PCR using degenerate oligonucleotides that randomized the TIRs. The TIR region of five arbitrary clones from each library were sequenced to validate diversity in the libraries. A total of 96 clones were randomly picked and cultivated. Cultures were back-diluted after overnight incubation, induced when required and expression levels were assayed after 5 h. The fluorescence levels varied greatly amongst the clones with the highest and the lowest *gfp*-expressing clones differing by up to 1000-fold (Fig. 2b, c). This observation confirms that this part of the TIR is an important determinant of expression levels in a broad range of bacterial species.

### Antibiotic-based selection of high-producing variants from TIR libraries

**Fig. 3:**
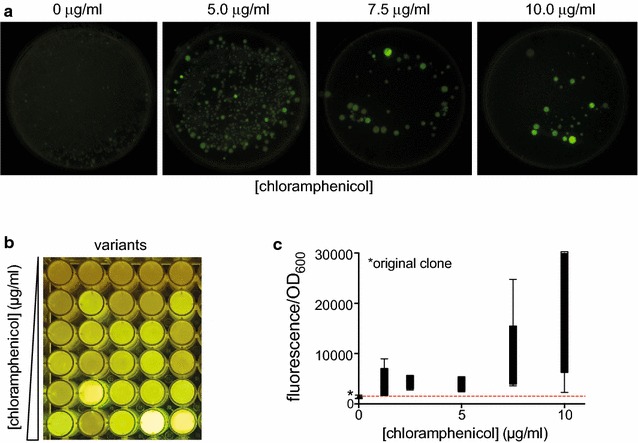
Antibiotic selection of maximal protein production in a Gram-positive bacterium. **a** *B. subtilis* TIR libraries expressing *gfp* were grown on agar plates with different chloramphenicol concentrations. GFP production levels on the different antibiotic concentrations were assayed by exposing agar plates to long-wave UV light. Five individual colonies were randomly picked from these plates and assayed for expression levels assessed by fluorescence per cell density in a microplate reader (**b**, **c**)

To select for the best TIRs, libraries were plated on solid media with increasing concentrations of chloramphenicol. The amount of colonies appearing on the plates decreased whereas the average fluorescence intensity increased with rising concentrations of antibiotic (Fig. 3a). Clones were randomly picked from the plates, recovered and grown overnight without antibiotic selection before measuring the fluorescence in a microplate reader (Fig. 3b). This analysis showed that the likelihood of isolating a highly fluorescent clone increased with increasing concentrations of antibiotics (Fig. 3c).

### Optimization of industrially relevant proteins

Finally we explored optimization of clones for production of different industrially relevant proteins in the two Gram-positive hosts. For *L. lactis* we chose to optimize expression of a tyrosine ammonia lyase (TAL) from *Flavobacterium johnsoniae*, which is expressed in the cytoplasm and converts tyrosine into *p*-coumaric acid in a single enzymatic step [29]. For *B. subtilis* we chose to target a *Micromonospora viridifaciens* sialidase (SIA), a hydrolase that cleaves the sialic acid residues of glycans [30]. In both cases well-established expression set-ups were used that were reported to result in high expression levels.

We first integrated the selection module hp-*Cm*^*R*^ downstream of the genes-of-interest into the plasmids used in the previous studies for the corresponding enzyme production [29, 30]. In these experiments, cytoplasmic expression of *tal* in *L. lactis* was controlled by the inducible P_nisA_ promoter in a pNZ8048 vector; however, we had to exchange the original chloramphenicol cassette of the vector backbone for an erythromycin resistance gene, to be able to first select for the transformed plasmid then utilize the *hp*-*Cm*^*R*^ device for selection of highly expressing variants. Expression of *sia* in *B. subtilis* was under control of the strong constitutive P_32_ promoter and secreted by the aid of a CGTase signal peptide encoded in the replicative pDP66K plasmid. In both experiments, we refer to the TIR in the original construct as the TIR^orig^.

**Fig. 4:**
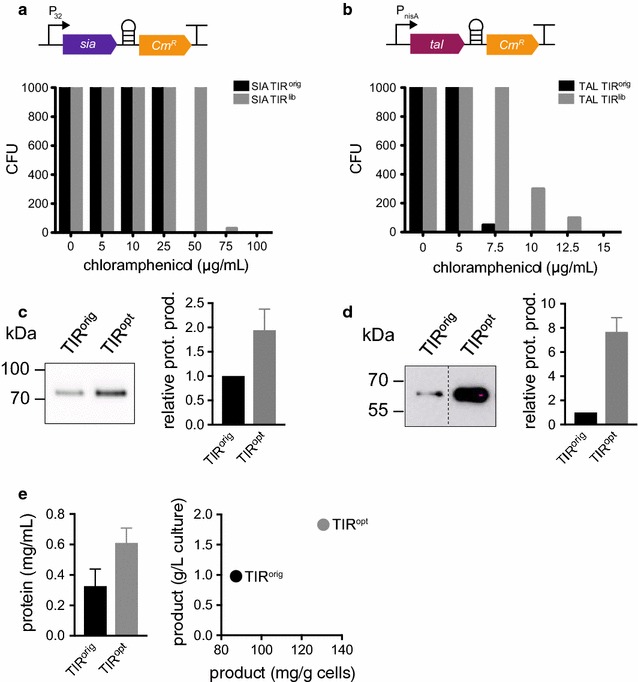
Production optimization of the industrially relevant proteins tyrosine ammonia lyase (TAL) and sialidase (SIA). **a** A His-tagged sialidase-encoding sequence was translationally coupled to the chloramphenicol resistance gene. A TIR library was constructed, transformed into *B. subtilis* and grown with different chloramphenicol concentrations on agar plates. Colony forming units (CFUs) were counted for all concentrations for the library (TIR^lib^) and the original, non-randomized clone (TIR^Orig^) as control. **b** A Strep-tagged *tal*-encoding sequence was translationally coupled to the chloramphenicol resistance gene. A TIR library was constructed, transformed into *L. lactis* and grown with different chloramphenicol concentrations on agar plates. CFUs were counted for all concentrations for the library (TIR^lib^) and the original, non-randomized clone (TIR^orig^) as control. **c** The sialidase production level in the culture supernatant of the best performing clone (TIR^opt^) was analyzed by Western blot, using an antibody against a His-tag (left panel). The increase in production was analyzed by densitometry and plotted as the relative protein production compared to the original clone (right panel). **d** TAL production level of the best performing clone (TIR^opt^) was analyzed by Western blotting using an antibody against Strep-tag (left panel). The increase in production was analyzed by densitometry and plotted as the relative protein production compared to the original clone (right panel). **e** Sialidase (TIR^opt^) was purified via a Ni^2+^-NTA column and purified product concentration was estimated using a BCA assay (left panel). Product per cell mass and per culture volume was calculated (right panel). The original construct (TIR^orig^) was used for comparison

The TIR^orig^ was then randomized by PCR using degenerate oligonucleotides, employing the same strategy as with the *gfp*-*hp*-*Cm*^*R*^ library. After transformation into the hosts and overnight recovery with the appropriate vector backbone antibiotics in liquid culture, both libraries were back-diluted, induced when required and after 5 h plated on solid media with different concentrations of chloramphenicol. We then determined the colony forming units (CFUs) of each library at different concentrations of chloramphenicol, and compared with the TIR^orig^ clones. In both cases we observed that the library produced more CFUs at higher chloramphenicol concentrations than the original construct (Fig. 4a, b). In addition, we observed a reduction in the amount of colonies appearing on the plates as the antibiotic concentration increased.

Colonies were recovered on 75 μg/mL chloramphenicol for *B. subtilis* and 15 μg/mL chloramphenicol for *L. lactis*. Protein levels were assessed by Western blotting using a His-tag antiserum for sialidase and a Strep-tag antiserum for TAL (Fig. 4c, d). In both cases, the clones isolated at the highest antibiotic concentration displayed a higher protein production compared to the TIR^orig^. Production of TAL in *L. lactis* was improved by approximately eightfold (Fig. 4d) and production of sialidase in *B. subtilis* was doubled (Fig. 4c) from approximately 1 to 2 g/L (Fig. 4e). In both cases, activity of the enzymes originating from the optimized TIR^opt^ variants was not compromised at the high level production (Additional file 1: Figure S1A, B).

## Discussion

A wide range of industrial applications use enzymes as a sustainable alternative to chemical catalysts. These applications include, but are not limited to, the manufacturing of food, paper, detergents and biofuels [31]. However refinement in production titres rely on enzyme discovery and gene and strain engineering, which are time consuming and labour intensive processes. Existing methods for high throughput screening of gene expression are based on e.g. droplet microfluidics or flow cytometry [18, 20]. Despite being extremely efficient and effective, these platforms are expensive to purchase and operate and their availability is limited to large-scale operations. Other screening methods rely on protein fusions, which might alter parameters such as expression, solubility and turnover rate [22]. To address this issue, we developed a simple growth based selection system based on translational coupling of an antibiotic resistance gene with the upstream gene sequence that leaves the protein of interest unaltered. Different from previous attempts, our system focuses on Gram-positive bacteria [25], works on genomically integrated targets and does not require tagging of the protein of interest [32]. Nonetheless, tags can be purposefully added to the protein if desired.

We first explored the relationship between gene expression and antibiotic resistance. A low correlation could indicate poor protein folding or poor melting of the mRNA hairpin. However, the correlation coefficient was above 0.9 in both hosts, which demonstrates that antibiotic sensitivity is tuneable and that the system responds linearly to the gene expression levels tested. Differences in the dynamic range of screenable chloramphenicol concentrations were observed between the two hosts; the smaller dynamic range in *B. subtilis* may be due to the initial choice of a poor-performing TIR for *gfp*. The expression was very low, even when controlled by a strong promoter. Once we built a combinatorial library randomizing the TIR, the best expressing variants improved in protein production by eightfold over the variant we used to characterize the mRNA hairpin.

Protein production constructs are often assembled in replicative or integrative plasmids. Previous work has shown that the junction created between the vector and the coding sequence can result in different levels of expression depending on e.g. the restriction site used [14]. The variability is not completely explained by the free energy (∆G) associated with mRNA folding and seems to be context specific, meaning that the cloning scar will affect the levels of expression in a manner that is not robustly computationally predictable. The creation of a TIR library circumvents the problem by optimizing the expression of a gene directly in the system in which it will be expressed.

Here we demonstrate the effectiveness of removing the cloning scar bias by improving the production of two industrially relevant proteins. We chose to optimize TAL in *L. lactis*, due to the wide range of compounds of biotechnological interest that are produced from the intermediate p-coumaric acid; these include for example the flavonoid naringenin or the stilbene resveratrol [11, 29]. For *B. subtilis* we optimized the production of a sialidase, as their enzymatic activity gained interest in relation to treatment of spinal cord injuries and there was a recent attempt to improve their activity and production using *B. subtilis* [30]. Both genes were cloned into vectors using traditional restriction enzyme based cloning and were already expressed with high yield. After creating a combinatorial library that modifies the TIR, we could select variants that produced two to eightfold more protein than the original clones. Most importantly, the sialidase production level in *B. subtilis* increased from 1 to 2 g/L in a 50 mL shake flask experiment, demonstrating that the specific TIR optimization and selection tool can achieve high yields, in the range of industrial titer levels, even when carried out in low density cultures. A substantial increase in yield is expected in industrial fed-batch fermentations.

The tool we developed has the potential to be transferred to other less amenable members of the *Bacillus* or *Lactococcus* families. With decreasing cost of DNA synthesis and the increasing interest in Gram-positive cell factories, different types of combinatorial libraries can be coupled with this selection system to screen for variants that produce a protein. Some examples are error prone PCR, promoter libraries, DNA shuffling or other in vitro and in vivo methodologies that generate genetic variability. The screening process is simple, inexpensive and can be carried out in a few days in any molecular biology laboratory and therefore presents an attractive alternative to advanced screening methodologies.

## Conclusion

We developed a selection-based system to screen for synthetically evolved TIRs in Gram-positive hosts. The system responds linearly to increasing concentrations of antibiotic by preventing growth of sub-optimal library variants. Using the selection tool, we demonstrate improved expression of industrially relevant proteins in two cell factory hosts, namely TAL in *L. lactis* and a sialidase in *B. subtilis*.

## Methods

### Bacterial strains, media and growth conditions

Bacterial strains used in this study are listed in Additional file 1: Table S1.

*Lactococcus lactis* strain NZ9000 ∆ *hsd* was used for all experimental procedures. Cells were grown at 30 °C in M17 broth (BD Difco, San Jose, CA, USA) supplemented with 1% glucose (GM17), without shaking. Electro competent cells were prepared as previously described [33]. Cultures were supplemented with 5 µg/mL erythromycin or 5 µg/mL chloramphenicol to select for plasmid presence, unless otherwise stated. Cultures were induced with 1.5 ng/mL nisin, unless otherwise stated.

*Bacillus subtilis* strain SCK6 (1A976 http://www.bgsc.org) was used for expression experiments. Cloning, library construction and propagation of plasmids were performed in *E. coli* NEB5α (New England Biolabs, Ipswich, MA, USA). *B. subtilis* was grown in lysogeny broth (LB) at 37 °C shaking (250 rpm). The antibiotics neomycin (5 µg/mL), kanamycin (50 µg/mL) and erythromycin (5 µg/mL) were supplemented when necessary. Transformation of integration vectors into *B. subtilis* was performed with chemically competent cells as previously described [34]. Correct genome integrations were confirmed by colony PCR and sequencing.

### Plasmids and strain construction

Plasmids and oligonucleotides used in this study are listed in Additional file 1: Tables S2 and S3. All constructs were made with uracil excision cloning as previously described [35].

Plasmids used in *L. lactis* are based on pNZ8048 from the NIsin Controlled gene Expression (NICE) system [26] and expression was controlled by the inducible nisin promoter. Translationally coupled versions were constructed using the standard chloramphenicol acetyltransferase resistance gene derived from pNZ8048 [26]. Hairpins were introduced by USER cloning using overlapping oligonucleotide tails coding for the hairpin [35, 36]. The resulting plasmids pNZ-*tal*-hp-Cm^R^ was built by adding the hp-Cm^R^ module to the pNZ_FjTAL described previously [29]. The plasmid pNZ-*gfp*-hp-Cm^R^ was built in two steps: first by cloning a GFP folding reporter [37] into pNZ8048, using primers 1 and 2. In a second step the module hp-Cm^R^ was added to the vector.

*Bacillus subtilis* plasmids were constructed in *E. coli* NEB5α (New England Biolabs, Ipswich, MA, USA), purified and subsequently transformed and integrated into *B. subtilis*. Integration vectors were based on the pDG268 plasmid, with integration in the *amyE* locus. Transcription was controlled by four different constitutive promoters of increasing strength: P_J23101_, P_liaG_, P_lepA_, and P_veg_ [27]. All four promoters variants were used to construct pDG-GFP-hp-Cm^R^. Promoters were amplified from the *B. subtilis* genome. The synthetic promoter pJ23101 was inserted into the plasmid using two overlapping oligonucleotides by PCR.

The replicative vector pDP66K-SIA-hp-Cm^R^ was constructed by adding the hp-Cm^R^ module to the plasmid pDP66K-Mv [30] which uses the promoter P32 to drive transcription.

### Library construction

For the construction of TIR libraries a degenerated forward oligonucleotide specific for the gene of interest was designed. The six nucleotides upstream of the start codon were changed to all possible combinations whereas the six nucleotides downstream the start codon were changes to all possible synonymous codons.

Libraries for *L. lactis* were constructed by amplification of the whole pNZ-derived plasmid using the degenerated forward oligonucleotide and a reverse oligonucleotide with a pairing USER cloning overlap. The plasmid library was built by amplifying the template plasmid containing *gfp*, *gfp*-hp-Cm^R^ or *tal*-hp-Cm^R^ with the degenerate oligonucleotides and circularized using USER cloning as described elsewhere [35]. Libraries were transformed directly into *L. lactis* with no intermediate steps. The reference construct for the *tal* gene, here referred to as the TIR^orig^ clone, was previously described [29].

Libraries for *B. subtilis* were constructed in *E. coli* MC1061 by amplification of the whole pDG268*neo* or pDP66K-Mv plasmids using degenerated forward oligonucleotides and reverse oligonucleotides sharing 15 nucleotide homology with the forward oligonucleotide. Q5 polymerase (New England Biolabs, Ipswich, MA, USA) was used to amplify the template plasmid containing *gfp*, *gfp*-hp-Cm^R^ or *sia*-hp-Cm^R^. Library construction was performed as described before [14]. The reference construct for the *sia* gene, here referred to as the TIR^orig^ clone, was previous described [30].

### Expression and selection

Expression of individual *L. lactis* clones, were assayed using overnight cultures prepared by inoculating a single colony in 5 mL GM17 supplemented with respective antibiotics and incubated at 30 °C without shaking. Cultures were then back-diluted (1:50) into 5 mL of GM17 media containing the appropriate antibiotics and incubated at 30 °C without shaking. At OD_600_ 0.3–0.6 cultures were induced with 1.5 ng/mL nisin and incubated for 3 h.

For the assessment of individual clones, *B. subtilis* overnight cultures were prepared by inoculating a single colony in 5 mL LB media supplemented with the appropriate antibiotics and incubated at 37 °C with shaking. Cultures were then back-diluted (1:50) into 5 mL of LB media containing the appropriate antibiotics and incubated at 37 °C with shaking for 5 or 23 h.

*Ca.* 1 μg of plasmid library was transformed into *L. lactis* or *B. subtilis* using standard protocols [33, 34]. Cells were recovered for 1 h after transformation in GM17MC or LB media, transferred to GM17 or LB media supplemented with antibiotics and grown overnight at 30 °C for *L. lactis* and 37 °C for *B. subtilis*.

*Lactococcus lactis* cultures were then back-diluted (1:50) into 10 mL GM17 media containing the appropriate antibiotics and incubated at 30 °C. Cultures were induced at OD_600_ 0.3–0.6 and after 5 h 0.2 OD units were plated on GM17 plates with increasing concentrations of chloramphenicol and incubated at 30 °C overnight.

After overnight incubation, *B. subtilis* cultures were back-diluted (1:50) into 5 mL LB media containing the appropriate antibiotics and incubated at 37 °C with shaking. 5 h after dilution, OD_600_ was measured and 0.2 OD units of cells were then plated on LB agar plates containing different concentrations of chloramphenicol and incubated overnight at 37 °C.

Selection was performed as previously described [36]. Selected expression variants were sequenced.

### MIC determination

For *L. lactis* MIC determinations, 5 mL GM17 media with 5 µg/mL erythromycin were inoculated with a single colony containing pNZGFP-hp-Cm^R^ and grown overnight at 30 °C. Cultures were then back-diluted (1:50) into 10 mL GM17 media supplemented with 5 µg/mL erythromycin, and their growth monitored. At OD 0.3 the culture was split into 8 different 2 mL eppendorf tubes and induced with different concentrations of nisin (0; 0.25; 0.5; 0.75; 1; 1.5; 5 or 10 ng/mL). Cultures were incubated at 30 °C for 2 h and 0.01 ODU of each culture was transferred to a 96 wells plate (Greiner, Kremsmünster, Austria) containing 200 μL GM17 media (ca. 5 × 10^6^ cfu/mL), and a serial dilution of chloramphenicol and respective concentrations of nisin as inducer. Plates were incubated for 15 h at 30 °C.

For *Bacillus subtilis* MIC determinations, 5 mL LB media were inoculated with 4 strains that constitutively expressed *gfp*-hp-*Cm*^*R*^ and grown overnight at 37 °C with shaking. Cultures were then back-diluted (1:100) into 5 mL LB media containing the appropriate antibiotic in a 24-deep well plate (EnzyScreen, Heemstede, Netherlands). Cultures were incubated at 37 °C with shaking. After 2 h, 10 μL of each culture was transferred to a 96 well plate (Greiner, Austria) containing 100 μL LB media (ca. 5 × 10^5^ cfu/mL) and a serial dilution of chloramphenicol. Plates were incubated for 18 h.

To assess translational coupling, fluorescence (Ex: 485 nm, Em: 516 nm for GFP) and OD_600_ were measured in an MX plate reader (Biotek, Winooski, VT, USA). The MIC value was defined as the lowest antibiotic concentration at which the final OD_600_ represented less than 10% of the entire population after background correction. Each MIC experiment was conducted with biological triplicates.

### Protein detection and quantification

Western Blot analysis was performed by resuspending *L. lactis* grown as described above in 50% volume of CelLytic B (Sigma Aldrich, St. Louis, MO, USA) supplemented with lysozyme, egg white (Amresco, Solon, OH, USA), benzonase nuclease (≥ 250 units/μL, Sigma Aldrich, St. Louis, MO, USA) and Roche cOmplete™ Protease Inhibitor Cocktail (Sigma Aldrich, St. Louis, MO, USA) and incubated for 1 h before the samples were sonicated. An aliquot of 20 µL of each sample was incubated for 1 h with 1 µL of a 1:10 dilution of CY5 dye (Amersham quick stain, GE healthcare, Chicago, IL, USA) for total protein quantification. The sample was then mixed with the same volume of 5 × reducing sample buffer and heated to 95 °C for 5 min for protein denaturation. 0.05 ODU of the samples were loaded onto a 4–20% Mini-PROTEAN-TGX gel (BioRad, Hercules, CA, USA) and run for 35 min at 175 V.

For *B. subtilis*, 10 μL of supernatant were mixed with 5 μL of 5× reducing sample buffer and heated to 95 °C for 5 min for protein denaturation. 10 μL of the samples were loaded onto a 4–20% Mini-PROTEAN-TGX gel (BioRad, Hercules, CA, USA) and run for 35 min at 175 V.

Proteins were transferred from the protein gel to a nitrocellulose membrane using the iBlot^®^ dry blotting system (Invitrogen, Thermo Fisher Scientific, Waltham, MA, USA) at 25 V for 7 min. The proteins were detected with the help of antigen-specific antibodies.

For *B. subtilis* sialidase an anti-His antibody (1:1000; Merck Millipore, Merck KGaA, Darmstadt, Germany) was used. The antibody was diluted in 5% w/v skim milk in TBS-T (20 mM Tris–HCl pH 7.6, 150 mM NaCl, 0.1% v/v Tween-20), the secondary antibody was diluted in TBS-T. For *L. lactis* an anti-Strep (1:10,000, Biorad, Hercules, CA, USA) directly coupled to HRP was used.

The HRP-coupled antibody was visualized using Amersham ECL Prime Western Blotting Detection Reagent (GE Healthcare, Chicago, IL, USA). The chemoluminescence signal was detected using a G:Box bioimager (Syngene, Cambridge, UK). The resulting images were analysed by densitometry using the Fiji software [38].

### Sialidase protein purification

50 mL LB broth supplemented with 50 µg/mL kanamycin was inoculated with an overnight culture of *B. subtilis* SCK6 transformed with pDP66K-SIA-hp-Cm^R^ to an OD_600_ of 0.05. Cells were grown for 23 h at 37 °C with shaking (250 rpm). After 23 h the supernatant was harvested by centrifugation at 5000×*g* for 15 min and 4 °C and passed through a 0.45 μm filter (Frisenette ApS, Knebel, Denmark) and a 0.20 μm filter (Sartorius AG, Göttingen, Germany). The filtered supernatant was then concentrated using a 10 K Amicon concentrator (Merck, Darmstadt, Germany), mixed with 10 mL purification buffer (50 mM NaH_2_PO_4_, 300 mM NaCl, 10 mM imidazole, pH 8.0) and again concentrated. The final concentrate was subjected to a Ni–NTA spin column (Qiagen, Hilden, Germany). The column was washed twice with 600 μL of washing buffer (50 mM NaH_2_PO_4_, 300 mM NaCl, 20 mM imidazole, pH 8.0) and finally protein was eluted twice with 300 μL elution buffer (50 mM NaH_2_PO_4_, 300 mM NaCl, 500 mM imidazole, pH 8.0). To reduce imidazole concentration several cycles of concentrating and diluting in storage buffer (20 mM NaH_2_PO_4_, 100 mM NaCl and 10% glycerol, pH 7.4) were performed using a 10 K Amicon concentrator (Merck, Darmstadt, Germany). The final volume was adjusted to 200 μL and protein concentration was estimated using BCA assay (Merck, Darmstadt, Germany). BSA was used as standard.

### Sialidase activity assay

To determine activity of the sialidase, supernatant and concentrated supernatant of expression cultures of the original and optimized clones and purified proteins were diluted in 50 mM phosphate-citrate buffer pH 7.0. The enzymatic reaction was started by addition of the substrate pNP-Neu5Ac (Sigma Aldrich, St. Louis, MO, USA) at a concentration of 0.75 mM in a 100 μL reaction. Absorbance at 410 nm was monitored continuously for 1 h in an MX plate reader (Biotek, USA). The reaction rate was calculated from the slope of the initial linear section of the curve.

### TAL activity assay

The TAL activity was measured as described by Jendersen and colleagues [29]. Briefly, expression of *tal* was induced with 1.5 ng/mL nisin in chemically defined media (CDM) as described above. Cultures were grown at 30 °C for 16 h, harvested at 13000 g and the supernatant was recovered. The concentration of p-coumarate was measured by HPLC using a gradient method with two solvents (0.1% ammonium formate and acetonitrile) and quantified measuring absorbance at 290 nm.

## Additional file

**Additional file 1.** Supplementary information for a synbio approach for selection of highly expressed gene variants in Gram-positive bacteria.

## Abbreviations

Cm: chloramphenicol
HRP: horse radish peroxidase
LB: lysogeny broth
NICE: NIsin Controlled gene Expression
OD_600_: optical density at 600 nm
SIA: sialidase
SD: Shine Dalgarno
TAL: tyrosine ammonia lyase
TIR: translation initiation region
