# Supplementary material for: A synbio approach for selection of highly expressed gene variants in Gram-positive bacteria
Source: Microb Cell Fact. 2018 Mar 8;17:37. doi: 10.1186/s12934-018-0886-y (PMC5842541; doi:10.1186/s12934-018-0886-y)
Supplement: Supplementary file 1 — Additional file 1. Supplementary information for a synbio approach for selection of highly expressed gene variants in Gram-positive bacteria. [file 12934_2018_886_MOESM1_ESM.pdf]

Additional file 1

Supplementary information for
A synbio approach for selection of highly expressed gene variants in Gram-positive bacteria

Roberto Ferro[1,2†], Maja Rennig[1†], Cristina Hernandez Rollan[1], Daniel O. Daley[3,4] and Morten H. H. Nørholm[1,4]

[1]Novo Nordisk Foundation Center for Biosustainability, Technical University of Denmark, 2800 Kgs. Lyngby, Denmark
[2]Department of Plant and Environmental Science, University of Copenhagen, 1871 Frederiksberg, Denmark
[3]Center for Biomembrane Research, Department of Biochemistry and Biophysics, Stockholm University, Stockholm, Sweden.
[4]CloneOpt AB, Upplands Väsby, Sweden

[†] Contributed equally

Address of correspondence: morno@biosustain.dtu.dk

This supplementary file contains:

Figure S1: Activity assessment of selected library variants
Table S1: Strains used in this study
Table S2: Plasmids used in this study
Table S3: Oligonucleotides used in this study

Figure S1 - Activity assessment of selected library clones

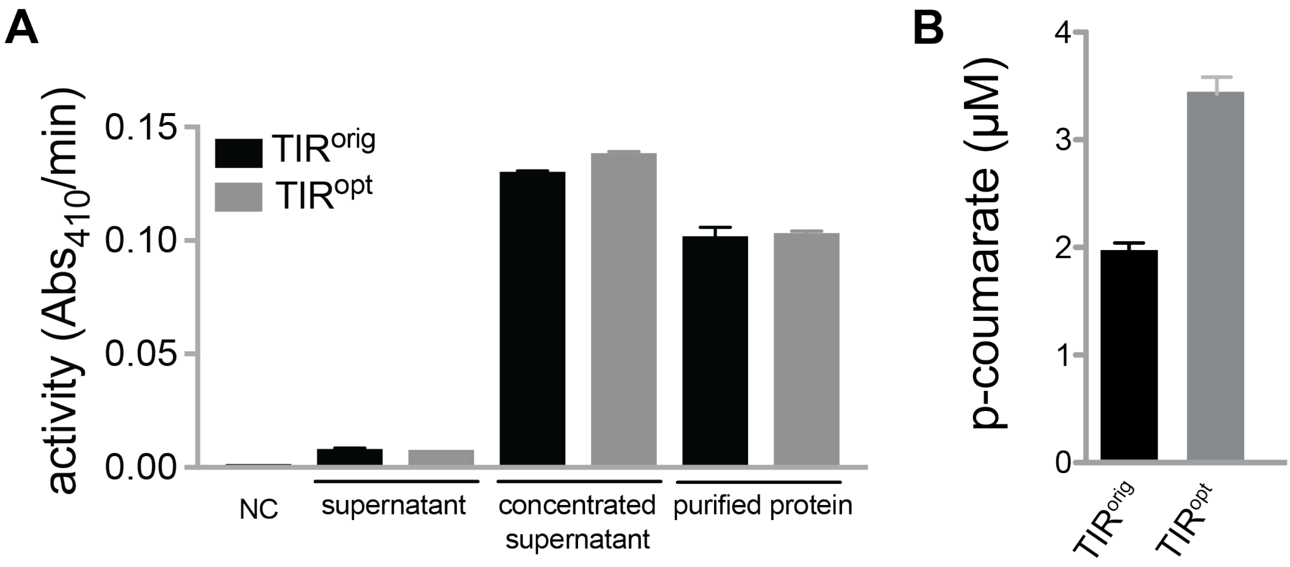

Figure S1: To assure that optimized variants are still active, activity assays for sialidase and TAL were performed. (A) Activity of the optimized sialidase clone was measured as absorbance at 410 nm per minute. Activity for culture supernatant, concentrated culture supernatant and purified protein were assessed. pNP-Neu5Ac was used as substrate. The negative control (NC) contained buffer and substrate but no enzyme was supplemented. (B) Activity of the optimized TAL clone was measured as product formation in µM by HPLC.

Table S1. Strains used in this study

Strain	Genotype	Source/Reference
L. lactis NZ9000 - Δ *hsd*	Δ *hsd, pepN::nisRK*	Dudnik, not published
B. subtilis SCK6	Em, his, nprE18, aprE3, eglSΔ102, bglT/bglSΔEV, lacA::PxylA-comK	[1]
B. subtilis SCK6 Pveg-*gfp*	Em, his, nprE18, aprE3, eglSΔ102, bglT/bglSΔEV, lacA::PxylA-comK, amyE::Pveg-gfp-hp-CmR	This study
B. subtilis SCK6 PlepA-*gfp*	Em, his, nprE18, aprE3, eglSΔ102, bglT/bglSΔEV, lacA::PxylA-comK, amyE::PlepA-gfp-hp-CmR	This study
B. subtilis SCK6 PliaG-*gfp*	Em, his, nprE18, aprE3, eglSΔ102, bglT/bglSΔEV, lacA::PxylA-comK, amyE::PliaG-gfp-hp-CmR	This study
B. subtilis SCK6 PJ23101-*gfp*	Em, his, nprE18, aprE3, eglSΔ102, bglT/bglSΔEV, lacA::PxylA-comK, amyE::PJ23101-gfp-hp-CmR	This study
E. coli NEB5α	fhuA2 Δ(argF-lacZ)U169 phoA glnV44 Φ80 Δ(lacZ)M15 gyrA96 recA1 relA1 endA1 thi-1 hsdR17	New England Biolabs, USA
E. coli MC1061	araD139, Δ(ara, leu)7697, ΔlacX74, galU-, galK-, hsr-, hsm+, strA	In house

Table S2. Plasmids used in this study

Plasmid	Property	Source/Reference
pNZ8048	*L. lactis* gene expression vector, Cm^R, P_{nisA}	[2]
pNZ_FjTAL	Pnz8048 vector with P_{nisA}-*tal* insert - Cm^R	[3]
pNZ-TAL-hp-Cm^R	Pnz8048 vector Erm^R, with P_{nisA}-*tal*-hp-Cm^R insert	This study
pNZ-GFP-hp-Cm^R	Pnz8048 vector, Erm^R, with P_{nisA}-*gfp*-hp-Cm^R insert	This study
pDG268-neo	Shuttle vector, ColE1 origin, Amp^R, MCS for integration into *amyE* locus with Neo^R	[4]
pDG-Pveg-GFP-hp-Cm^R	Shuttle vector, ColE1 origin, Amp^R, for integration of P_{veg}-gfp-hp-Cm^R into *amyE* locus	This study
pDG-PlepA-GFP-hp-Cm^R	Shuttle vector, ColE1 origin, Amp^R, for integration of P_{lepA}-gfp-hp-Cm^R into *amyE* locus	This study
pDG-PliaG-GFP-hp-Cm^R	Shuttle vector, ColE1 origin, Amp^R, for integration of P_{liaG}-gfp-hp-Cm^R into *amyE* locus	This study
pDG-PJ23101-GFP-hp-Cm^R	Shuttle vector, ColE1 origin, Amp^R, for integration of P_{J23101}-gfp-hp-Cm^R into *amyE* locus	This study
pDP66K-Mv	Cloning and expression vector, Km^R, encodes P32-sp-*sia*	[5]
pDP66K-SIA-hp-Cm^R	Cloning and expression vector, Km^R, encodes P32-sp-*sia*-hp-Cm^R	This study

Table S3. Oligonucleotides used in this study

Name	Sequence (5' --> 3')
**Cloning oligonucleotides**	
2995_pDGneo_fwd	AAAAGCAUTAGTGTATCAACAAGC
2989_pDGneo_rev	AGCTATTUCAGCTGCGCTTTTTCCATTATGTACTATTTCGATCAGAC
2993_CmR_fwd	ATAGGAGGUCCTCCTatgTCAaactttaataaaattgatttagacaattg
2994_CmR_rev	AAATAGCUGCGCTTTTTTGTGTCATAAttataaaagccagtcattaggc
2999_pVeg_fwd	ATGCTTTUGGAGTTCTGAGAATTGGTATG
3000_pVeg_rev	ACAGTAGUACTACATTTATTGTACAACACGAGC
3001_pLepA_fwd	ACAGTAGUACTATTAAACGCAAAATACACTAGC
3002_pLepA_rev	ATGCTTTUAGTCAATGTATGAATGGATACGG
3003_pLiaI_fwd	ACAGTAGUTCGTTTTCCTTGTCTTCATCT
3004_pLiaI_rev	ATGCTTTUATTGGCCAAAGCAGAAAG
3005_pLiaG_rev	ATGCTTTUCAAAAATCAGACCAGACAAAAG
3006_pLiaG_fwd	ACAGTAGUTCATTCATTCTATTATAAAGGAAAAGC
3044_pJ23101_fwd	ACAGTAGUgctagcataatacctaggactgagctagctgtaaaGGATCCTAGAAGCTTATCGA
3135_ GFPsf_rev	ACCTCCTAUGTCAttTgtatagttcatccatgcc
3136 _GFPsf_fwd	AGGatgcgUaaaggagaagaactt
3137_pDG_CmR_rev	ATAGGAGGUCCTCctatgtcaa
3138 _pDG_Pveg_fwd	acgcatCCUCGAGcctcctA
3206_pDG_pVeg-GFP-lib_fwd	ACTACTGUaggaggCNNNNNNatgcgNaaRggagaagaacttttcactgg
3474_pDP66K_fwd	AGGATCCUGCCTGCGAT
3475_sia-His_rev	ACCTCCTAUGTCAATGATGGTGGTGATGG
3476_Term_CmR_rev	AGGATCCUAAAAAGCGCAGCTGAAA
3489_pDP66K-P32-Sia-lib_fwd	ACGATTACATAGGAGGNNNNNNATGAARAARTTTCTGAAATCGACAGCTGC
3490_pDP66K-lib_rev	CTCCTATGTAATCGTTTGAATTCCAGGCTTGTCCGCTGTCGCCGGATCCC
1_GFPfr_fwd	actcaccaUGTCCAAAGGAGAAGAACTT
2_GFPfr_rev	aacCTTTGUAGAGCTCATCCATGC
3a_hpCmR_rev	ACCTCCTAUGTCATTTTTCAAATTGTGGATGGC
3_CmR_fwd	ATAGGAGGUCCTCCTatgTCAaactttaataaaattgatttagacaattg
4_CmR+histag_rev	ATGATGAUGGGCCGCAAGCTTtaaaagccagtcattaggcctatc
4b_Backbone_HIS_fwd	ATCATCAUCACCACCACCACCACtaatcaattgaaatggcaattaaac
5_RND-GFPfr_fwd	ataaattaUaaggaggcactcaccATGTCNAARGGAGAAGAA

	CTTTTCACTGG
6_RND-GFP-fr_rev	ATAATTTAUTTTGTAGTTCCTTCG
7_ TAL_Strep_fwd	ATCCACAAUTTGAAAAATAAtctagagagctcaagctttct
8_ TAL_Strep_rev	ATTGTGGAUGGCTCCAGCTTTTAGAACCattgttaatcag gtggtcttttac
9_ ERMSwap_fwd	attgaaUGCTTCAGTTGTCTTATTTCTAGATCT
10_ERMSwap_rev	atctcataUTATTTCCTCCCGTTAAATAATAGATA
11_ BB-ERM_rev	attcaaUaatccctcctctca
12_ BB-ERM_fwd	atatgagaUaatgccgactgt
13_TALRndlib_fwd	ATAAATTAUAAGGAGGCANNNNNNatgAAYACNatcaa cgaatatctgagcc
14_TALRndlib_rev	ATAATTTAUTTTGTAGTTCCTTCG

**Sequencing oligonucleotides**	
3052_AmyE_SeqS_rev	TGCCTGAACGAGAAGCTAT
3051_AmyE_SeqL_rev	TATATAAACCATTTAGCACGTAATCA
2990_pDGseq_rev	TGTATCAAGATAAGAAAGAACAAGTTC
3064_AmyE_SeqL_fwd	ATGTTTGCAAAACGATTCA
3015_pDGseq_fwd	CCAATGAGGTTAAGAGTATTCC
3528_P32_seq_fwd	gatatgataagattaatagt
3529_Sialidase_seq_rev	GTTGGGCGGCCGTCGTATGA
3530_KanR_seq_fwd	aagcctgattgggagaaaat
TalSeq_fwd	gattaccattgttcaggcg
TalSeq_rev	acaaaccggactcagcg
FW_insert_seq	actaacctgccccgttagt
RV insert seq	ATTCCTTGGUCCTTTAATTGGTGGACA

References supplementary material

1. Zhang X, Zhang YP. Simple , fast and high-efficiency transformation system for directed evolution of cellulase in Bacillus subtilis. 2011;4:98–105.

2. Kuipers OP, De Ruyter PGGA, Kleerebezem M, De Vos WM, Ruyter P, Kleerebezem M, et al. Quorum sensing-controlled gene expression in lactic acid bacteria. J Biotechnol. 1998;64:15–21.

3. Jendresen CB, Stahlhut SG, Li M, Gaspar P, Siedler S, Förster J, et al. Highly active and specific tyrosine ammonia-lyases from diverse origins enable enhanced production of aromatic compounds in bacteria and Saccharomyces cerevisiae. Appl. Environ. Microbiol. 2015;81:4458–76.

4. Jers C, Kobir A, Søndergaard EO, Jensen PR, Mijakovic I. Bacillus subtilis Two-Component System Sensory Kinase DegS Is Regulated by Serine Phosphorylation in Its Input Domain. PLoS One. 2011;6(2):e14653.

5. Jers C, Guo Y, Kepp KP, Mikkelsen JD. Mutants of micromonospora viridifaciens sialidase have highly variable activities on natural and non-natural substrates. Protein Eng. Des. Sel. 2015;28:37–44.
